# Protocol for AI-based prediction of problematic digital technology use among Indian youth: a Centre for Advanced Research on Addictive Behaviours initiative

**DOI:** 10.3389/fpsyg.2026.1754046

**Published:** 2026-03-17

**Authors:** Yatan Pal Singh Balhara, Rajeev Ranjan, Siddharth Sarkar, Simran Kaur, Ragul Ganesh, Shivanand Kattimani, Vandena Saxena, Subhash Das, Vishal Dhiman, Rachna Bhargava, Tanmay Joshi, Anindo Majumdar, Swarndeep Singh, Bichitra Nanda Patra

**Affiliations:** 1 Behavioral Addictions Clinic (BAC) and Centre for Advanced Research on Addictive Behaviours (CAR-AB), National Drug Dependence Treatment Centre (NDDTC), All India Institute of Medical Sciences (AIIMS), New Delhi, India; 2 All India Institute of Medical Sciences, Patna, India; 3 AIIMS, New Delhi, India; 4 Department of Physiology, All India Institute of Medical Sciences (AIIMS), New Delhi, India; 5 Jawaharlal Institute of Postgraduate Medical Education & Research (JIPMER), Puducherry, India; 6 CIE, Department of Education, University of Delhi, New Delhi, India; 7 Department of Psychiatry, North Eastern Indira Gandhi Regional Institute of Health and Medical Sciences (NEIGRIHMS), Shillong, India; 8 All India Institute of Medical Sciences, Rishikesh, India; 9 All India Institute of Medical Sciences, Bhopal, India; 10 Vardhman Mahavir Medical College (VMMC), Safdarjung Hospital, New Delhi, India; 11 Department of Psychiatry, AIIMS, New Delhi, India

**Keywords:** addiction, AI-based predictive model, anxiety, depression, digital addiction, machine learning, problematic technology use, youth mental health

## Abstract

**Background:**

Artificial intelligence (AI) and machine learning have an important role in mental health research by helping to predict and prevent digital addiction and problematic digital technology use. These include behaviors linked with internet, smartphones, gaming, social media, gambling, over-the-top (OTT) platforms watching, pornography watching, shopping/ buying, and excessive screen time.

**Objective:**

This multi-center study aims to develop and validate an AI-based predictive model to identify Indian youth at risk of problematic use of digital technology and associated psychological outcomes such as stress, anxiety, depression, and addiction. The study corresponds to one of the objectives of the Centre for Advanced Research on Addictive Behaviours (CAR-AB) initiative.

**Methods:**

Students aged ≥12 years from schools and colleges across six Indian sites (New Delhi, Bhopal, Patna, Puducherry, Rishikesh, and Shillong) will be recruited. Data will be collected on demographic, psychological, behavioral, cognitive, socio-environmental, and digital phenotype correlates using validated instruments. Machine-learning models, including ensemble and deep-learning methods, will be trained, validated, and interpreted using explainable AI techniques.

**Expected outcomes:**

The study will develop a validated and interpretable predictive model for early detection of vulnerability to problematic use of digital technology. Findings are expected to inform targeted interventions, guide digital-wellness policies, and support the integration of predictive tools within educational, work, and community settings.

## 1 Introduction

Problematic use of digital technology has emerged as a significant public health concern worldwide. This is particularly true for the youth including children, adolescents and young adults (Kuss and Lopez-Fernandez, 2016; Hawi and Samaha, 2017; Domingues-Montanari, 2017; Burkauskas et al., 2022).

Problematic use of digital technology refers to a pattern of technology engagement marked by impaired control, growing priority over other activities, and continued use despite negative outcomes. It may be associated with distress, dysfunction, or both. This pattern can occur through online or offline modes and across multiple contexts. These include internet use, gaming, smartphone use, social media use, gambling, shopping or buying, pornography watching, OTT content watching, excessive screen time, and related digital behaviors (Balhara et al., 2025b). The World Health Organization now recognizes certain digital technology-related behaviors as diagnosable disorders (ICD, 2019). This highlights their clinical and psychosocial significance. These potentially addictive behaviors are associated with heightened risk of stress, depression, anxiety, suicidal ideation, impaired cognitive performance. These may be associated with deterioration in social, emotional, academic and occupational functioning.

In India, the youth are disproportionately affected by these issues due to increasing digital penetration and limited regulatory oversight. A meta-analysis indicated that the prevalence of internet addiction among adolescents attending school in India is 24.1% with moderate cases accounting for 21.5% (95% CI: 17.0–26.8%) and severe cases comprising 2.6% (95% CI: 1.6–4.2%) (Joseph et al., 2022). Another meta- analysis on the prevalence of internet addiction among college students in the Indian setting reported the prevalence to be 19.9% (95% CI: 19.3 to 20.5%) (Joseph et al., 2022).

The Economic Survey of India (2024–2025) emphasized this concern. It noted that overuse of the internet and social media contributes significantly to mental health issues among children and adolescents. This highlights the urgent need for systematic, evidence-based, and scalable interventions focused on prevention, early detection, and management of problematic digital technology use (Ministry of Finance, Government of India, 2025).

Addictive behaviors have been identified as an emerging area in the broader context of addictions in India (Balhara et al., 2025a; Balhara et al., 2018; Kumar et al., 2019). The Centre for Advanced Research on Addictive Behaviours (CAR-AB) was conceptualized as a national level, implementation-focused initiative to address these gaps through a transdisciplinary approach (Balhara et al., 2025a). CAR-AB aims to develop evidence- based tools, interventions and resources to address the problematic use of digital technology and associated stress, anxiety, depression and addiction. While the youth is at the core of the initiative, it is believed that the outputs of the initiative shall be relevant to broader demographic profiles within the country and abroad.

Traditional research has established foundational epidemiological insights into addictive behaviors. In more recent times developments in artificial intelligence (AI) and machine learning (ML) have presented opportunities for predictive modeling in behavioral health. Studies across Asia, Europe, and Latin America (Lee and Kim, 2021; Kim et al., 2024; Giraldo-Jiménez et al., 2022; Gan et al., 2025; Perrot et al., 2022) have demonstrated the feasibility of ML algorithms in identifying individuals at risk of smartphone or internet addiction (Table 1).

**Table 1:** Studies utilizing AI-based predictive models to identify vulnerability to problematic digital technology use.

Sl No.	Study title	Author, year, Country	Sample	AI model used	Results (accuracy/validity)
1	Prediction of Problematic Smartphone Use: A Machine Learning Approach	Lee and Kim (2021); South Korea	Data was collected from 29,712 Native South Koreans (14,790 male and 14,922 female) between 3–60 years of age, out of which people below the age of 40 were taken as sample.	Decision tree, Random Forest, and XGBoost algorithm	The accuracy rate was the highest for the Random Forest (82.59%) model and the lowest for the Decision Tree Model (74.56%).
2	Explainable prediction of problematic smartphone use among South Korea’s children and adolescents using a Machine learning approach	Kim et al. (2024); South Korea	The data used in this study was collected from the Smartphone Overdependence Survey conducted by the National Information Society Agency between 2017 and 2021.	Logistic Regression, Random Forest, Gradient Boosting Machine (GBM), Extreme Gradient Boosting (XGBoost), Light GBM, Categorical Boosting,	The XGBoost predicted 87.60% participants at risk of developing potential problematic smartphone use.
3	Smartphones dependency risk analysis using machine-learning predictive models	Giraldo-Jiménez et al. (2022); Colombia	A sample of 1,247 students from undergraduate programs of a private university in Cali, Colombia. Randomly Stratified Sampling Technique was used.	Random Forest, logistic regression, and Support Vector Machine (SVM)	Random Forest, Logistic Regression and Support Vector Machine predicted smartphone dependency with an accuracy of 76–77% in young adults.
4	Application of machine learning in predicting adolescent Internet behavioral addiction	Gan et al. (2025); China	A total of 4,461 high school students in Chongqing, China were selected using stratified cluster sampling,	Six methods— Multi-level Perceptron, Random Forest, K-nearest Neighbor, Support Vector Machine, Logistic Regression, and eXtreme Gradient Boosting were used to construct the model.	The performance in predicting adolescent internet addiction behavior is average, with the extreme gradient enhancement method performing better than other models.
5	Development and validation of a prediction model for online gambling problems based on players’ account data	Perrot et al. (2022); France	Two random samples of French online gamblers in skill-based (poker, horse race betting and sports betting, n = 8,172) and pure chance games (scratch games and lotteries, n = 5,404) were taken	Predictive models for gambling with mixed validity.	The first model demonstrated good predictive performance while the second model showed moderate performance as it need to be validated with a larger dataset to ensure reliability. It could help in intervention programs of highly vulnerable gamers.
Indian research					
6	Machine Learning Model for Prediction of Smartphone Addiction	Raj et al. (2024); India	This research study has used the openly available dataset of smartphone usage by people.	A combination of machine learning algorithms such as Decision Tree, Logistic Regression, and Random Forest were used to analyze smartphone addiction	Random Forest algorithm achieved the best accuracy with a score of (0.89), the decision tree algorithm achieved the accuracy score of (0.86). The least performer was Logistic Regression which achieved an accuracy score of (0.74).
7	Trapped in the Mobile Screen: A Machine Learning Approach to Predict Nomophobia	Tapas et al. (2025); India	The study comprised 255 students who had been using their phones for at least one to two hours per day	They used 4 ML models namely Naive Bayes (NB) Classifier, K-Nearest Neighbors (KNN), Multi-Layer Perceptron (MLP), and Support Vector Machine (SVM) to predict nomophobia in 255 college going students in Jaipur, India.	SVM emerged as the most effective classifying algorithms with almost perfect accuracy, precision, and recall rates.

Despite the growing burden of digital technology-related addictions and their impact on youth, there is a lack of robust and accurate predictive models to identify individuals at risk in India. There are limited such data-driven models in the country (Raj et al., 2024). This precludes efforts for timely identification and early prevention of problematic use of digital technology. There is an urgent need to develop Artificial Intelligence (AI) algorithms with high accuracy to predict vulnerability to problematic use of digital technology and associated stress, anxiety, depression and addiction within the Indian population. The current study leverages the infrastructure and interdisciplinary collaborations of CAR-AB (Balhara et al., 2025a). The aim is to bridge the gap by developing and validating a contextually grounded, explainable AI model tailored to Indian youth.

The current study is in direct response to one of the objectives of the CAR-AB initiative. It aims to create an AI-based predictive model to detect vulnerability to excessive and problematic use of digital technology among youth. The model shall be guided by demographic, psychological, cognitive, behavioral, socio-environmental, and digital phenotype correlates.

### 1.1 Objective

The study aims to build and test an AI model that can identify youth at risk for problematic use of digital technology and associated stress, anxiety, depression and addiction. The final goal is to produce a tested model that can guide early screening and support future steps in prevention across school, college, health and community settings. The primary outcome of the study will be a composite risk level indicating vulnerability to problematic use of digital technology. This will be derived from validated cut-offs on behavioral addiction scales. In addition, secondary analyses will develop separate predictive models for key subtypes of problematic digital technology use. These will include smartphone use, internet use, social media use, gaming, gambling, etc.

## 2 Methods

### 2.1 Ethics procedure

The study obtained approval from the institutional ethics committee prior to commencement at the respective sites. Informed assent/consent will be obtained from the participants and, informed consent from parents or legal guardians will be obtained for minors. Participants will have the right to withdraw their assent/consent during the study without providing any justification. No personally identifiable information will be gathered. For digital phenotyping only the minimum necessary data will be collected. The collected data will be safely kept and preserved. Anonymization techniques will be used to prevent any potential re-identification of the participants. The research report will not disclose the identity of the participants.

### 2.2 Proposed study design, participants, setting

The study will utilize a cross-sectional analytical, observational study design. Participants will include students of school and colleges aged 12 years and above. School participants will be recruited from three different academic levels including middle stage (7th grade), secondary stage (8th to 10th grade) and senior secondary stage (11th and 12th grade). The college participants will be chosen from diverse academic backgrounds, including both undergraduate and postgraduate courses. The study will be conducted at six locations across the country for pan-national representation. These shall include New Delhi, Bhopal (Madhya Pradesh), Patna (Bihar), Puducherry, Rishikesh (Uttarakhand), and Shillong (Meghalaya). Data will be collected from schools and colleges located in rural and urban areas.

#### 2.2.1 Inclusion criteria

Participants will include school and college students aged 12 years and above. Students must be able to read and respond to the study tools, provide assent or consent, and have parental consent if they are minors. Both rural and urban institutions will be eligible for inclusion in the study.

#### 2.2.2 Exclusion criteria

Students who do not provide assent or consent, or whose parents do not provide consent in the case of minors, will be excluded. Any student who is unable to participate due to school or personal constraints will also be excluded.

### 2.3 Sampling technique and sample size estimation

#### 2.3.1 School students

To ensure wider representation, the sample will be distributed among the six study sites. A list of schools expressing interest in participating in the study will be compiled for each of the sites. A random selection process will be used where a computer-generated method will be employed to draw a proportionate random sample to recruit students taking into account their grade and gender representation from the list of eligible students at each site. The same strategy will be employed across the middle stage (7th and 8th grade), secondary stage (9th and 10th grade) and the senior secondary stage (11th and 12th grade). The school students shall be recruited in three sets of grades including middle stage, secondary stage and the senior secondary stage. Schools from both rural and urban areas shall be included.

A meta-analysis indicated that the prevalence of internet addiction among adolescents attending school in India is 24.1% with moderate cases accounting for 21.5% (95% CI: 17.0–26.8%) and severe cases comprising 2.6% (95% CI: 1.6–4.2%) (Joseph et al., 2022). Assuming that 24% of the individuals in the population are likely to have the condition in question, the study would require a sample size of 281 to estimate the expected proportion with an absolute precision of 5% and a 95% confidence level. A minimum of 281 students will be enrolled from each educational stage including middle, secondary, and senior secondary. This approach will result in a total minimum sample of 843 school students.

#### 2.3.2 College students

To ensure wider representation, the sample will be distributed among the six study sites. Colleges located in both urban and rural areas will be covered. A list of colleges expressing interest in participating in the study will be compiled. A random selection process will be used where a computer-generated method will be employed to draw a proportionate random sample to recruit students taking into account their stream and gender representation from the list of eligible students at each site.

A prior meta- analysis on the prevalence of internet addiction among college students in the Indian setting reported the prevalence to be 19.9% (95% CI: 19.3 to 20.5%) (Joseph et al., 2021). Expecting that 19% of the subjects in the population are expected to have the factor of interest, the study would require a sample size of 237 to estimate the expected proportion with 5% absolute precision and 95% confidence level.

### 2.4 Variables for which data will be collected

The data shall be collected on various demographic, psychological, cognitive, behavioral, socio- environmental and digital phenotype correlates of excessive and problematic use of digital technology and associated stress, depression, anxiety, and addiction. These shall include structured proforma and instruments as mentioned in Supplementary Table S1.

#### 2.4.1 Structured proforma for socio- demographic profile

A structured proforma shall be used to collect the details about the socio- demographic and socio- environmental profile of the participants. This shall include the age, gender, level of education, field of study, year of study, household income level, type of residence (urban, rural, suburban), educational qualification of parents, occupation of parents, living arrangement, and other relevant variables.

#### 2.4.2 Structured proforma for details of use of digital devices and internet

A structured proforma shall be used to collect the details about the use of digital devices and the internet.

#### 2.4.3 Study instruments

Various study instruments shall be used to collect the details about psychological, cognitive, behavioral, socio- environmental and digital phenotype correlates of excessive and problematic use of digital technology and associated stress, depression, anxiety, and addiction. Digital phenotype correlates will be captured as aggregated indicators of digital technology use. These will include overall screen-time exposure across major device types, patterns of primary and background screen use, and temporal distribution of use. No continuous monitoring, content data, communication logs, or location data will be collected or stored. The list of questionnaires or instruments to be applied to measure these correlates has been provided in Supplementary Table S1.

### 2.5 Statistical analysis plan

This study aims to develop and validate an AI-based predictive model to identify children, adolescents and young adults at high risk of developing problematic digital technology use (internet, smartphones, gaming, social media, gambling, shopping/ buying, Over the Top (OTT) content watching, pornography watching, and excessive screen time). This process involves collecting information from students, cleaning and organizing it, and then using machine learning tools to build and test a model that can identify early signs of problematic use. The model’s accuracy will be checked carefully and simple explanation tools will ensure it is transparent and trustworthy. Table 2 enumerates the key steps of AI-predictive model development and validation for the detection of vulnerability to problematic digital technology use from our study population.

**Table 2:** Key steps of AI-predictive model development and validation for detecting vulnerability to problematic digital technology use.

S.No	Components/key steps	Process	Key activities	Purpose
1	Pre-processing	Cleaning, transformation, standardization, and handling missing values/outliers.	Data cleaning and normalization; removal of duplicate or noisy data, imputation for missing values, standardization and scaling of variable	Prepare high-quality, structured data suitable for analysis and modeling.
2	Exploratory data analysis (EDA)	Descriptive statistics, correlations, and PCA.	Descriptive summaries (mean, SD, range), correlation heat maps and scatter plots, Principal Component Analysis (PCA) for dimensionality reduction	Identify patterns, relationships, and trends in data.
3	Feature selection	Statistical and machine learning–based selection of key predictors.	Use of statistical tests, Recursive Feature Elimination (RFE), LASSO regression to select most appropriate predictive variables	Retain the most relevant and impactful features for the model.
4	Model building	Construction of predictive algorithms.	Implement ML and DL models (regression, classification, ensemble, and neural networks), use Generative AI (GANs) to generate synthetic data for robustness.	Build the core AI-based predictive model for detecting vulnerability to problematic digital technology use.
5	Validation	Model Evaluation and performance testing	Apply K-Fold Cross Validation, assess metrics: MAE, MSE, Accuracy, Precision, Recall, F1-score	Evaluate accuracy, reliability, and generalizability of the model.
6	Interpretation and monitoring	Model explainability and performance tracking.	Use SHAP (Shapley Additive Explanations) and LIME (Local Interpretable Model-Agnostic Explanations), implement drift detection for continuous monitoring of data shifts	Ensure interpretability, transparency, and long-term model stability.

The effective number of predictors will be reduced prior to modeling. This will enable stable model training, Stratified k-fold cross-validation will be used. This shall be done to ensure that each fold contains an adequate number of high-risk cases. This approach supports reliable estimation of performance for tree-based models, ensemble methods, and neural networks.

#### 2.5.1 AI-based data processing and analysis

**Figure 1:**
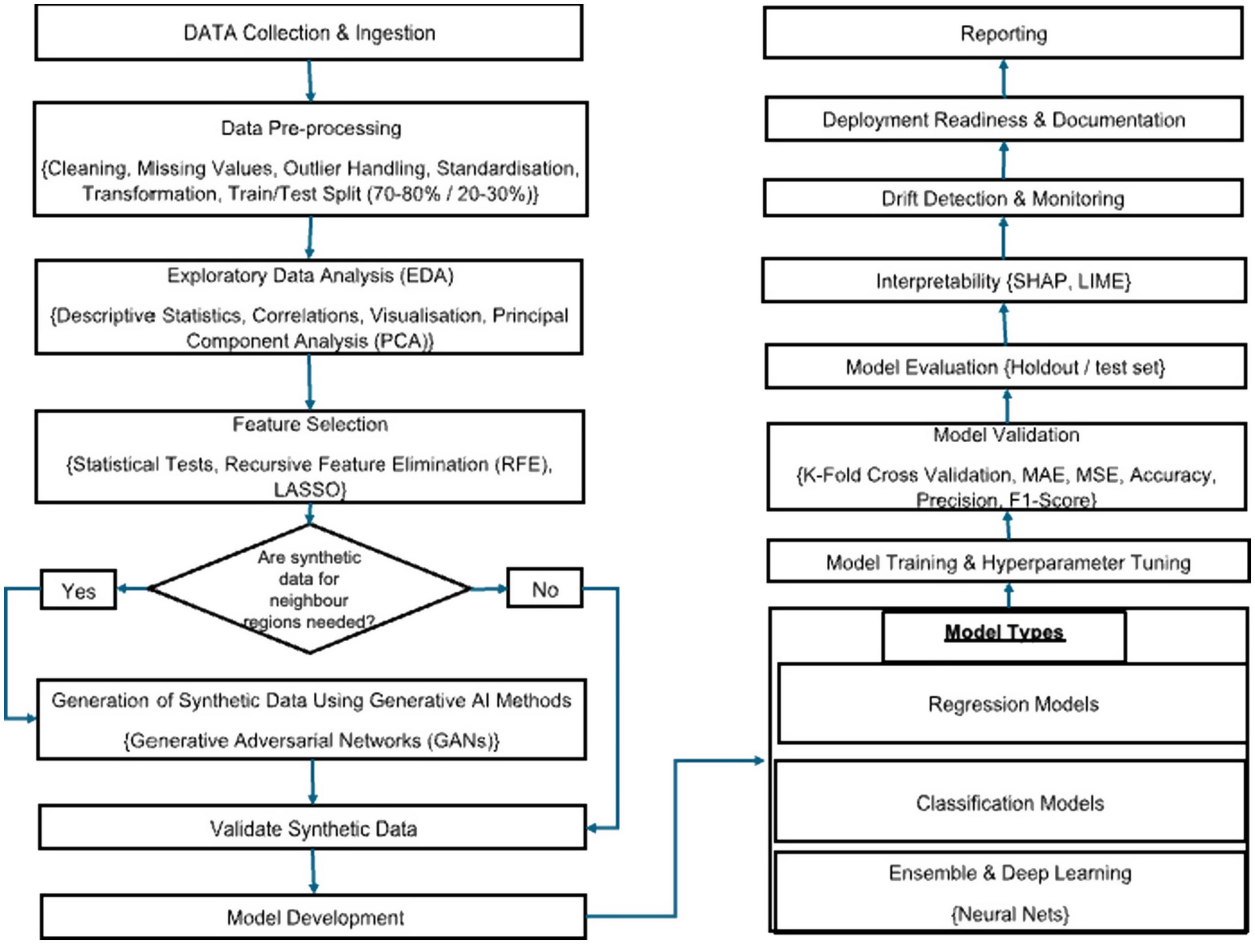
The flowchart depicting statistical analysis plan.

**Figure 2:**
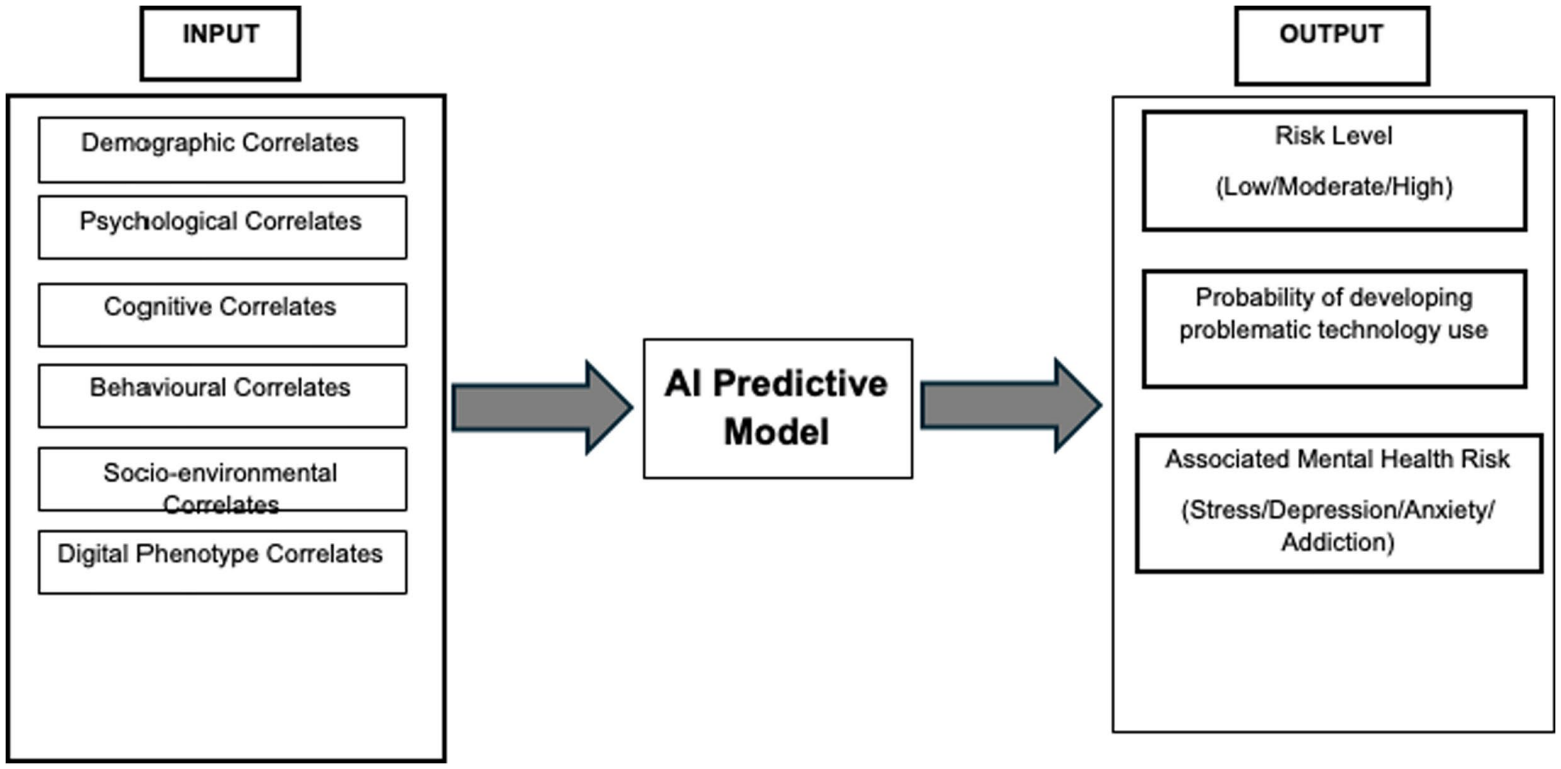
Conceptual framework depicting implementation plan of the proposed study.

The study will use an AI-driven analytic workflow to build a predictive model. The model will draw on behavioral, psychological, cognitive, social, and digital-use data. The steps for this workflow have been summarized in Figures 1, 2.

##### 2.5.1.1 Data processing

Data will be checked for errors, cleaned, and stored in a uniform format. Continuous variables will be scaled and inspected for extreme values. Categorical variables will be coded in a simple binary format. The data shall be evaluated for suitability for filling missing entries. If needed, the missing entries will be filled with tested methods including multiple imputation for scales, KNN for behavior data, and mixed-type imputation for combined fields. Outliers will be flagged with both numeric rules and isolation-based models so that rare but valid patterns are not removed.

##### 2.5.1.2 Exploratory analysis

Descriptive checks will be done to assess the range and spread of each variable. PCA and related tools will be used to see broad patterns in the data. Clustering methods will help sort users into groups that show distinct forms of digital use, such as heavy gaming, high social-media use, or broad multi-platform use. These results will guide later steps in selecting and shaping predictors.

##### 2.5.1.3 Feature selection

Predictors will be chosen with a mix of simple tests and machine-based tools. RFE, lasso, and mutual-information scores will help retain variables that add clear value to the model. Derived measures such as ratios from cognitive tasks or sums of key behaviors will be included when they add clarity.

##### 2.5.1.4 Model building

Several models will be trained in parallel. These will include logistic regression, boosted tree models, random forests, SVMs, and neural networks. Ensemble models will also be tested. Synthetic samples may be created with a controlled GAN-based method to avoid bias. This will be done in case the final dataset has uneven class sizes, each model will be trained with tuned parameters and checked for stable performance.

##### 2.5.1.5 Model testing

All models will be validated with cross-validation. The main performance measures will include accuracy, recall, precision, F1 score, AUROC, and error rates. Misclassified cases will be examined to see which traits most often lead to error and whether those errors come from specific subgroups.

##### 2.5.1.6 Explainable-AI review

SHAP and LIME explanations will be used to show how each feature affects the output. Partial-dependence plots will help show the direction and strength of these effects. This step will allow the model to be understood and used by the end users.

##### 2.5.1.7 Model stability checks

The model will be assessed for drift across sites and age groups. Drift-detection methods will check whether the link between predictors and outcomes changes. These checks will prepare the model for future field use.

Figure 1 depicts the analysis plan.

### 2.6 Implementation plan

The study will begin after obtaining the ethical clearance from the respective institutions involved. Afterwards, the schools and colleges interested in participation will be selected and they will be provided with consent and assent forms after briefing them about the research and its purpose. The school/college students providing consent and assent (with parental consent) will then be selected for participation in the study. Data collection shall follow this. Data analysis and model development shall be carried out subsequently. Kindly Refer to Figure 2 for the conceptualization framework of the implementation plan of our study.

### 2.7 Data management and monitoring

All data will be handled with a secure process. Each site will enter data into a coded database (using REDCap) with no personal identifiers. Records will be checked at the point of entry for missing fields, errors, and inconsistencies. A central team will review weekly uploads from all sites to ensure uniform coding, consistent variable labels, and correct scoring of all study tools. Any errors identified during this review will be sent back to the site for correction.

Only approved members of the study team will have access to the full dataset. Each site will keep a separate record of assent and consent forms, and these will not be linked to the main dataset.

Monitoring will include regular checks for outliers, and unusual response patterns. These checks will ensure that the dataset remains stable and suitable for AI model training. A central monitor will track recruitment numbers, data quality, and protocol adherence. Sites will report any deviation from procedures at once. All steps in data handling will follow good research-practice standards and adhere to the requirements of the ethics approvals granted for the study.

## 3 Results

We have presented the study protocol here and the results are not yet available. The results shall be published at a later stage.

## 4 Discussion

Prior research from India by our group has documented high prevalence and heterogeneous correlates of problematic internet and digital technology use among school and college students (Balhara et al., 2015; Balhara et al., 2018; Kumar et al., 2019; Balhara et al., 2021). The present study builds on this evidence by integrating these multi-domain correlates within a predictive modeling framework.

The present study represents one of the first large-scale, multi-center initiatives in India to integrate behavioral, psychological, cognitive, socio-environmental, and digital phenotype data for developing an AI-based predictive model of vulnerability to problematic use of digital technology and associated stress, anxiety, depression, and addiction among youth. This work is expected to make a critical empirical and methodological foundation for data-driven prevention, screening, and intervention in the domain of addictive behaviors.

This study proposes to advance the field by proposing a structured, multi-domain framework for predicting vulnerability to problematic use of digital technology among youth. The prior work is based largely on single-domain measures or linear models. The present protocol aims to integrate behavioral, psychological, cognitive, socio-environmental, and digital phenotype data. This design allows the model to capture complex risk patterns.

A key strength of the proposed approach is the emphasis on model interpretability. Such outputs can support early screening, guide targeted prevention strategies, and improve acceptance of AI-based tools among educators, public health experts, clinicians, and policymakers.

From a theoretical perspective, the proposed model intends to be in sync with dimensional and transdiagnostic frameworks of behavioral addiction (Brand et al., 2019; Billieux et al., 2015; Kardefelt-Winther et al., 2017). These frameworks emphasize graded risk. The use of explainable AI allows these theoretical constructs to be examined empirically. This will help strengthen links between addiction theory and applied prediction.

### 4.1 Expected outcomes

The study is expected to develop and validate a comprehensive AI-based predictive model for early detection of vulnerability to problematic use of digital technology and associated stress, anxiety, depression and addiction among youths. The model will integrate demographic, psychological, cognitive, behavioral, socio-environmental, and digital phenotype variables. This shall offer a multidimensional and data-driven understanding of risk and protective patterns. The use of deep learning and generative AI is expected to enhance predictive accuracy and generalizability. From a service delivery perspective, the model will enable the timely identification of at-risk individuals. This will facilitate early intervention and prevention of associated mental health concerns such as addiction, anxiety, depression, and stress.

### 4.2 Implications for public health and policy

The findings of this study are expected to contribute to digital-wellness policy and early-intervention frameworks by identifying individuals at risk, and the predictors of enhanced risk. Globally, there has been an increasing focus on regulating the access to potentially addictive behaviors. Ban on access to social media for adolescents under 16 years of age in Australia and introduction of The Promotion and Regulation of Online Gaming Bill, 2025 in India are some recent examples (Fardouly, 2025; PIB, 2025). The initiatives focused at targeted vulnerability assessment have the potential to shape the regulatory frameworks and offer more nuanced solutions. India’s youth constitute over one-third of its population. They are particularly susceptible to the psychological and social consequences of problematic digital technology engagement. The AI-based predictive model proposed here can serve as a screening and triaging tool within schools, colleges, workplace and community settings to identify individuals at elevated risk and facilitate targeted preventive interventions. This shall add to the existing universal prevention-focused interventions targeted at youth in the country (Balhara et al., 2023).

These data also have implications for policy. The regulation of online gaming and gambling in India remains fragmented (Singh and Balhara, 2024; Balhara, 2024; Seth and Balhara, 2024; Balhara et al., 2025b) Also, despite calls to have contextually relevant guidelines on potentially additive behaviors and screen time, limited progress has happened (Singh and Balhara, 2021). Predictive analytics can guide evidence-based regulation and help delineate boundaries between healthy digital engagement and problematic use. This aligns with the National Education Policy and National Mental Health Policy. Additionally, creation of such tools aligns with the Government of India’s National Digital Health Mission, Ayushman Bharat School Health and Wellness Programme (AB-SHWP), Rashtriya Kishor Swasthya Karyakram, the National Mental Health Programme, Digital India Programme, the IndiaAI Mission, the National Youth Policy, and Samagra Shiksha Abhiyan.

### 4.3 Ethical considerations

Developing predictive models for youth behavior raises certain ethical concerns. These include data privacy, potential stigmatization, and algorithmic bias. In this study, use of data minimization, anonymization, and interpretability mechanisms shall be incorporated to ensure transparency and explainability. This is in keeping with international recommendations for ethical deployment of AI in mental health (Warrier et al., 2023). The multi-site design further enhances generalizability. The study protocol has been approved by the institutional ethics committee at all the participating sites. Parental consent and students’ assent in case of minors and consent in case of the participant aged 18 years or older shall be obtained.

### 4.4 Anticipated challenges

Several challenges are anticipated during the implementation of this study. There may be delays in participant recruitment due to the need for institutional approvals from schools and colleges. In addition, the need for obtaining parental consent shall also require additional time. The need for parental consent might also impact the response rate in case of minors. Accurate collection of screen-time and digital behavior data is likely to be a major challenge. This is anticipated due to privacy concerns. This may also introduce selection bias if more cautious parents and students opt out. The cross-sectional design allows identification of only vulnerability associations. Incorporating advanced AI models may pose methodological and logistical difficulties. These include risks of synthetic bias if generative algorithms are not properly trained.

### 4.5 Status and timeline of the study

The data collection has begun at all six sites. The data collection is expected to be completed over the course of the next 6 months.

## 5 Conclusion

This study intends to leverage AI to predict vulnerability to problematic use of digital technology and associated stress, anxiety, depression and addiction among Indian youth. This is in keeping with the CAR-AB’s goal to advance evidence-based science on addictive behaviors. It intends to develop a scalable framework for early identification and prevention of potentially addictive behaviors. The integration of empirical data, ethical AI, and public-health relevance intends to promote digital wellness and mental health resilience in India’s rapidly evolving technological landscape.

## Data Availability

The original contributions presented in the study are included in the article/Supplementary material, further inquiries can be directed to the corresponding author/s.
