# Supplementary material for: Protocol for AI-based prediction of problematic digital technology use among Indian youth: a Centre for Advanced Research on Addictive Behaviours initiative
Source: Front Psychol. 2026 Mar 17;17:1754046. doi: 10.3389/fpsyg.2026.1754046 (PMC13036647; doi:10.3389/fpsyg.2026.1754046)
Supplement: Supplementary file 1 [file Table_1.docx]

| **Supplementary Table S1: Study Questionnaires/ instruments to measure multi-dimensional correlates** | | | | |
| --- | --- | --- | --- | --- |
| **S.No.** | **Name of Instrument** | **Construct to be Measured** | **Response Format** | **Purpose** |
| **Behavioural Correlates** | | | | |
| 1 | Smartphone Addiction Scale – Short Version (SAS–SV) | Problematic smartphone use | 6-point Likert | Measures daily life disturbance, withdrawal, overuse, tolerance, etc. |
| 2 | Internet Addiction Test (IAT) – 6 item | Internet addiction | 5-point Likert | Assesses IA’s impact on functioning and academic performance |
| 3 | Bergen Social Media Addiction Scale (BSMAS) | Social media addiction | 5-point Likert (1–5) | Measures preoccupation, tolerance, withdrawal, and negative impact |
| 4 | MULTICAGE CAD-4 | Addictive behaviors (substance & behavioral) | Yes/No | Screens for multiple addictions and impulse-control disorders |
| 5 | Internet Gaming Disorder Scale – Short Form (IGDS9-SF) | Internet gaming disorder | 5-point Likert (1–5) | Measures 9 DSM-5 IGD criteria |
| 6 | Gaming Disorder and Hazardous Gaming Scale (GDHGS) | Gaming disorder (ICD-11) | Clinician-rated | Screens for ICD-11-based gaming disorder and impairment |
| 7 | Motives for Online Gaming Questionnaire (MOGQ) | Motives for online gaming | 5-point Likert | Measures seven gaming motives (Escape, Coping, Social, etc.) |
| 8 | Brief Biosocial Gambling Screen (BBGS) | Problematic gambling | Yes/No | Rapid screen for disordered gambling |
| 9 | Screen Time Questionnaire | Device-based screen time | Self-report categories by device | Quantifies time spent on screens (TV, smartphone, etc.) |
| 10 | Insomnia Severity Index – 2 | Insomnia symptoms | 5-point Likert | Rapid screening for insomnia symptoms |
| **Psychological Correlates** | | | | |
| 11 | Barratt Impulsiveness Scale – Brief (BIS–Brief) | Impulsivity (attentional, motor, non-planning) | 4-point Likert | Measures impulsive personality traits |
| 12 | Brief Sensation Seeking Scale | Sensation seeking | 5-point Likert | Assesses need for novel/stimulating experiences |
| 13 | Brief Aggression Questionnaire | Aggression (physical, verbal, anger, hostility) | 5-point Likert | Measures multidimensional aggression |
| 14 | Brief Rosenberg Self-Esteem Scale (B-RSES) | Self-esteem | 4-point Likert | Short, valid measure of global self-esteem |
| 15 | Fear of Missing Out Scale (FoMOS–sf) | Fear of missing out | 5-point Likert | Quick screening of FOMO tendencies |
| 16 | Relationship Questionnaire (RQ) | Adult attachment style | 7-point Likert | Measures secure, avoidant, anxious, dismissing styles |
| 17 | Emotional Subscale of Interpersonal Orientation Scale | Emotional support / affiliation motivation | 5-point Likert | Correlates with related social support measures |
| 18 | Perceived Stress Scale (PSS–4) | Perceived stress | 5-point Likert | Measures perceived stress appraisal |
| 19 | Ten Item Personality Inventory (TIPI) | Big Five personality traits | 7-point Likert | Quick measure of Big Five personality traits |
| 20 | Three-Item Loneliness Scale (TILS) | Loneliness / social isolation | 3-point scale (1–3) | Brief measure of subjective loneliness |
| 21 | Visual Analogue Scale (VAS) for Body Image Issues | Body dissatisfaction | 100-point VAS | Measures dissatisfaction with appearance, figure, etc. |
| 22 | WHO Well-Being Index (WHO-5) | Psychological well-being | 6-point Likert (0–5) | Screens for depression and measures mental well-being |
| **Socio-environmental Correlates** | | | | |
| 23 | Unmet Need to Belong | Need for belongingness | 5-point Likert | Measures perceived fulfillment of emotional needs |
| 24 | Social Support (Objective & Subjective) | Social support | Mixed response types | Measures network size, proximity, satisfaction |
| 25 | Inadequate Parental Supervision | Parental involvement | Categorical / Likert | Measures parental engagement & work status |
| 26 | Similar Peer Group Behavior | Peer influence in smartphone use | Likert / categorical | Problematic use of technology peer pressure and normalization of use |
| **Neuro-cognitive Correlates** | | | | |
| 27 | Trail Making Test (TMT) | Attention, processing speed, executive function | Performance/time-based | Measures cognitive flexibility and visual-motor speed |
| 28 | Emotional Stroop Test | Attentional bias to emotion | Reaction time | Measures attention toward emotional stimuli |
| 29 | Clock Drawing Test (CDT) | Visuospatial and executive functions | Drawing-based | Screens for cognitive impairment |
| 30 | Go/No-Go Task | Impulse control / response inhibition | Reaction time | Measures inhibitory control and attention |
